# Experimental Investigation of Concrete Runway Snow Melting Utilizing Heat Pipe Technology

**DOI:** 10.1155/2018/4343167

**Published:** 2018-02-07

**Authors:** Fengchen Chen, Xin Su, Qing Ye, Jianfeng Fu

**Affiliations:** ^1^China Airport Construction Group Corporation, Beijing, China; ^2^Beijing Super-Creative Technology Co., Ltd., Beijing, China

## Abstract

A full scale snow melting system with heat pipe technology is built in this work, which avoids the negative effects on concrete structure and environment caused by traditional deicing chemicals. The snow melting, ice-freezing performance and temperature distribution characteristics of heat pipe concrete runway were discussed by the outdoor experiments. The results show that the temperature of the concrete pavement is greatly improved with the heat pipe system. The environment temperature and embedded depth of heat pipe play a dominant role among the decision variables of the snow melting system. Heat pipe snow melting pavement melts the snow completely and avoids freezing at any time when the environment temperature is below freezing point, which is secure enough for planes take-off and landing. Besides, the exportation and recovery of geothermal energy indicate that this system can run for a long time. This paper will be useful for the design and application of the heat pipe used in the runway snow melting.

## 1. Introduction

In China, more than 100 airports meet with heavy snow or frozen ice in winter, which causes brake failing or accidents easily [1–3]. For avoiding accidents, most airports close runways and use the deicing chemicals [4] or machines [5–7] to remove snow. However, these methods need a large amount of manpower, materials, and machines, especially for the snow at night. Hence, flights usually have been delayed or even canceled. In addition, deicing chemicals have been proved to be harmful to pavement, vegetation, and water [8–10].

Wang et al. studied the concrete damage of five different deicing chemicals and test results showed that the two calcium chloride solutions caused the most damage [11]. The chemical properties of the vegetation with and without deicing chemicals have been sampled, and comparison results showed that chlorine (Cl) and sodium (Na) increased but phosphorus (P) and magnesium (Mg) decreased by the deicing chemicals [12]. Besides, the PH in the deicing chemicals vegetation was also significantly higher than that of the normal so that the vegetation cannot grow up healthily. What is more, the Geographic Information System (GIS) data in the county of Vastmanland (in Sweden) showed that the deicing salt, on the whole, accounts for more than half of the total chloride load in both groundwater and surface water [13]. Due to these defects of the deicing chemicals, efficient and environmental snow melting method needs to be researched particularly.

Innovative snow melting technologies can be classified using ground source heat pipe [14], heated fluids with heat pump [15–17], and electrical heaters [18, 19]. Among these methods, the heat pipe snow melting system has been regarded as the most environmental friendly method, as the ground heat source is used only which is a clean and renewable energy. The heat pipe snow melting system can transport the heat of the ground soil to pavement surface and melt snow anytime when the ground evaporator is warmer than the condenser imbedded in pavement [20, 21].

The heat pipe snow melting system was first used in a bridge deck in Trenton, New Jersey, in 1969 [22]. Ammonia has been used as the working fluid in the heat pipe because it is not susceptible to freezing and chemically inert with steels. The applied results show that the temperature of the heated deck surface was warmer than the normal portion of the bridge about 4°C, which was sufficient to antifreezing and snow melting in that place. Another project was constructed on a highway ramp in Wyoming. Even though the temperature of the ground was only 8.3°C, the heated surface increased to more than 0°C. Hardly heat pipe snow melting system project was built in China. An unsteady simulative model of geothermic heat pipe snow melting system was built by Zhang et al. [23]. Simulative results show that this system is efficient to improve the pavement surface temperature and melt snow as the ground temperature is supposed to be 15°C.

The above projects indicate that the performance of the heat pipe snow melting is various in different regions as the temperatures of the environment and ground soil are different; hence, the applicability of the system needs to be concerned. A full scale snow melting system test platform is developed in Beijing which references the foreign and domestic experience. The temperatures of pavement surface and ground soil are obtained based on the test platform. The influences of the air temperature and embedded depth in this system are analyzed. Besides, the different types of practical snow melting processes are researched in this paper, which could provide useful guides for proving the high reliability of the heat pipe snow melting system.

## 2. Experimental Investigation

### 2.1. Experimental Set-Up

**Figure 1:**
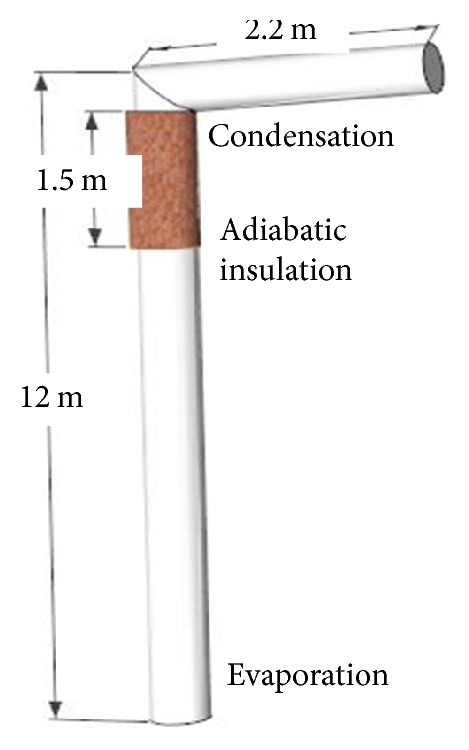
Heat pipe structure diagram.

Figure 1 shows a simplified diagram of the two-phase closed heat pipe. It mainly consists of a vertical evaporation section, adiabatic insulation, and a horizontal condensing section. The phase-change working fluid in the heat pipe is ammonia and the material of the pipe is carbon steel. The concrete mix proportions are as follows: 0.41 (water) : 1 (cement) : 1.15 (sand) : 2.45 (coarse aggregate). The compressive strength of concrete is 50.6 MPa, and the rupture strength is 5.6 MPa.

**Figure 2:**
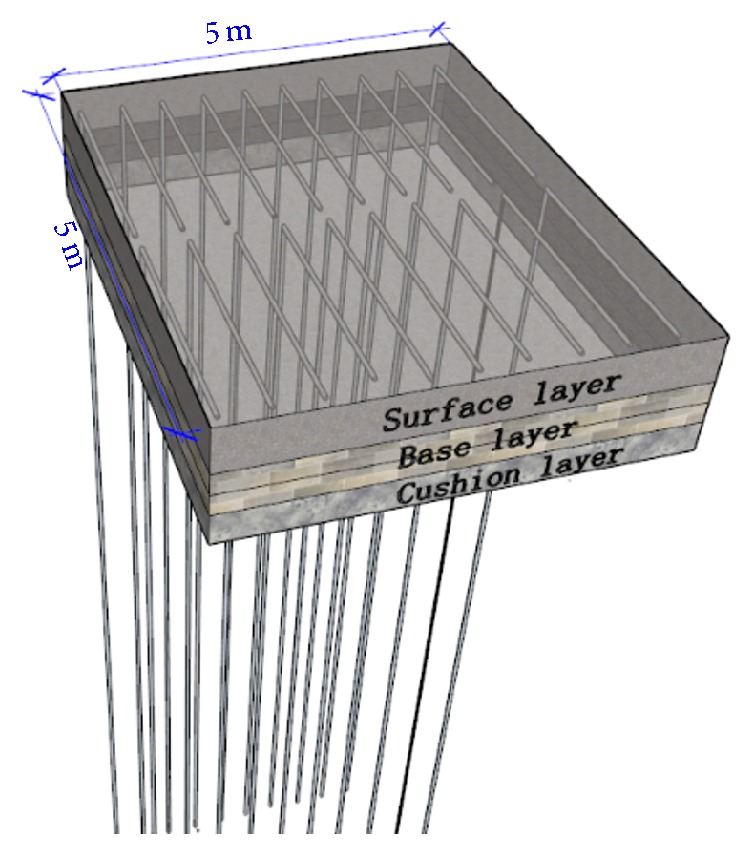
Heat pipe snow melting system.

The snow melting system based on the heat pipe technology is shown in Figure 2. In this system, the condensation of the heat pipe is embedded in the pavement, and the condensation is in the underground soil. The size of pavement surface layer is 5 m × 5 m × 0.4 m (length × width × thickness). The vertical and horizontal length of the pipe are 12 m and 2.2 m, and the diameter is 32 mm. For easy liquid backflow the pipe horizontal section has 1-degree upward angle. Considering the snow melting uniformity, the pipe spacing is 0.3 m. And the burial depth is 0.06 m and 0.15 m of two different concrete slabs.

### 2.2. Data Monitoring

**Figure 3:**
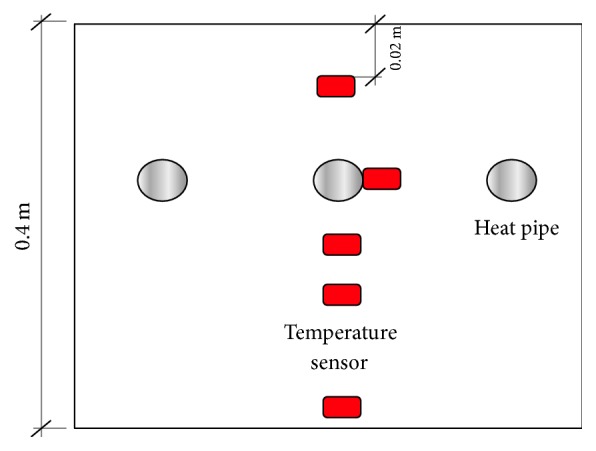
Temperature monitoring.

As shown in Figure 3, the temperature of the heat pipe pavement and the normal pavement in different depths has been measured by a series of temperature sensors. The resistance thermometer sensor was calibrated with the freezing point of water to 60°C boiling water to ensure the measurement accuracy. The calibration result shows that the temperature sensor in the system has small error.

**Figure 4:**
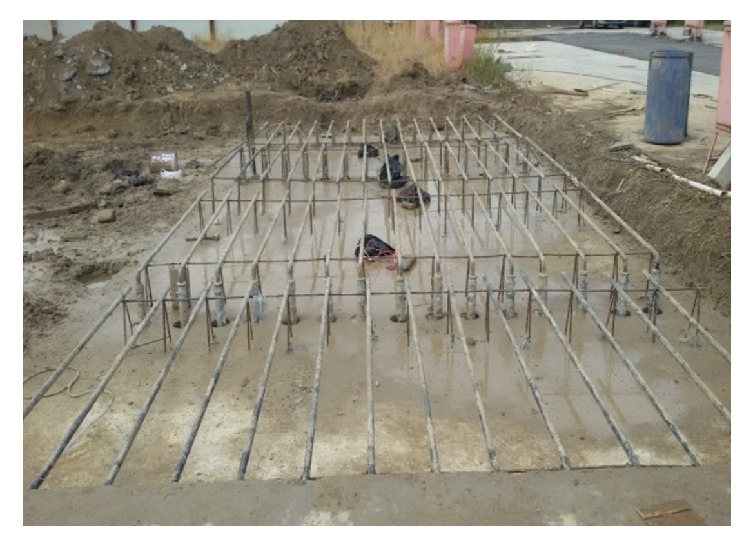
Full scale experimental platform.

The positions of these sensors are above or below the heat pipe, and the depth is from 0.02 m to 0.4 m, respectively. Using these sensors, we can clearly know the heat transfer of the heat pipe in the pavement. And the full scale system is shown in Figure 4.

## 3. Experimental Results and Discussion

### 3.1. Normal Pavement Temperature Distribution

**Figure 5:**
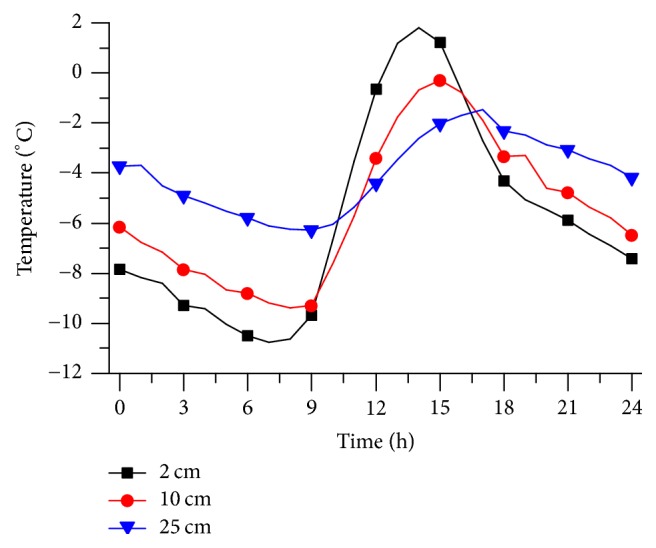
Normal pavement temperature at different times.

Figure 5 shows the temperature of normal pavement at different time of 12 January 2016 in Beijing, and the average air temperature is −8°C. From 0:00 to 24:00, the pavement temperature increased at 7:00 to 14:00 and decreased at the remaining time. The lowest temperature appeared at about 7:00, but the highest temperature usually appeared at about 14:00. The temperature curve on one day follows the negative sine wave, and it is regular for various depths. For 2 cm depth, the lowest temperature is −11°C, while the highest temperature has already exceeded 1°C. It means that the daily range of the pavement temperature is more than 12°C and the pavement has experienced freeze-thaw cycle. The surface has the risk of freezing when it rains, which is harmful to pavement and human.

**Figure 6:**
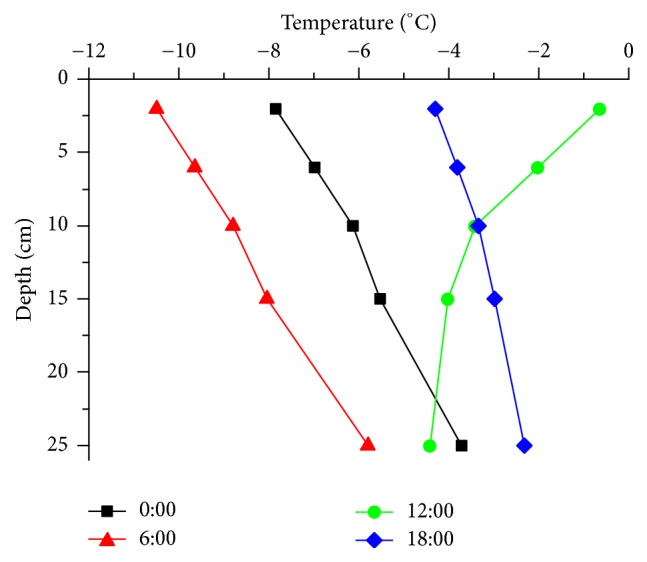
Normal pavement temperature in different depths.

Figure 6 further shows the pavement temperature varies with the depth. The temperature increased with the depth at most time with weak solar radiation. For example, with the pavement temperature at 6:00, the surface is lower than 25 cm, about 5°C. The reason for this phenomenon is that the outside air is colder than the pavement in winter, so the air conveys cooling to pavement; then the pavement is colder. As the cooling is conveyed from the surface to inside, the pavement temperature change ranges are decreased with depth; the surface of pavement is the most severely affected area by outside environment. On the contrary, the solar radiation is strong at noon, and the pavement temperature decreased with the depth.

### 3.2. Heat Pipe Pavement Temperature Distribution and Influence Factors

**Figure 7:**
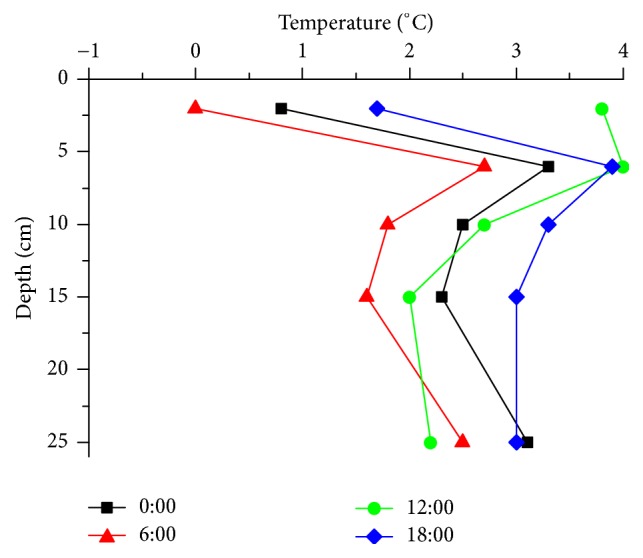
Heat pipe pavement temperature at different depths.

Figure 7 shows the heat pipe pavement temperature varies with the depth. As the heat pipe was embedded at 6 cm depth, the pavement temperature suddenly changes at this depth. Compared with the normal pavement in Figure 6, the 6 cm depth temperature increased by more than 10°C, and the 2 cm depth temperature increases by 8°C. The pavement surface temperature was up to 0°C all the time, which indicated that this system can melt snow and avoid freezing on the whole day. The daily temperature range of the heat pipe pavement was less than half of the normal pavement. It means that the heat pipe system can improve the stability of the pavement temperature.

**Figure 8:**
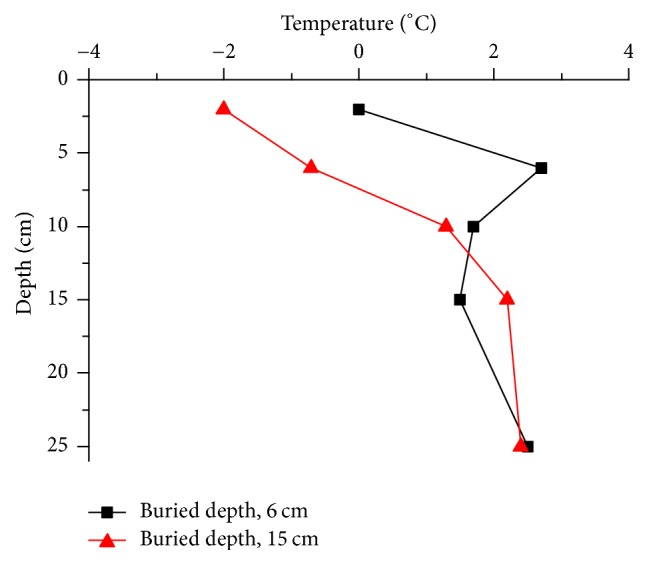
Impact of heat pipe embedded depth on pavement temperature field.

Figure 8 compares the temperature of pavements with different pipe embedded depths. Clearly, the surface temperature decreased by about 2°C as the pipe embedded depth increased from 6 cm to 15 cm. And the surface temperature is below the freezing point in the 15 cm burial depth slab which is dangerous for vehicles or pedestrians. However the temperature at 15 cm is higher in the 15 cm burial depth slab than the 6 cm burial depth slab. Therefore the temperature field can be improved by a proper burial depth of heat pipe.

**Figure 9:**
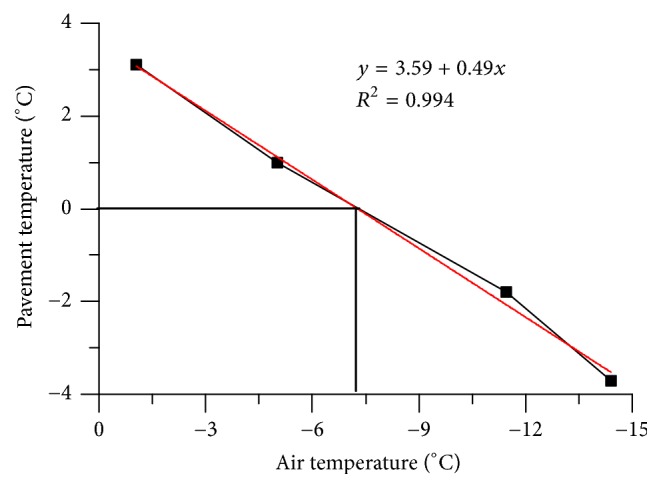
Impact of air temperature on pavement surface temperature.

Figure 9 shows the pavement surface temperature with different air temperatures. It can be seen that the surface temperature decreased with the decrease of the air temperature. And the relationship between air temperature and pavement surface temperature is nearly linear. At the same time, the pavement surface will be frozen point as the air temperature reaches −7.3°C; hence, it is better not to use this system at extremely cold areas.

### 3.3. Heat Pipe Pavement Snow Melting and Antifreeze Performance

**Figure 10:**
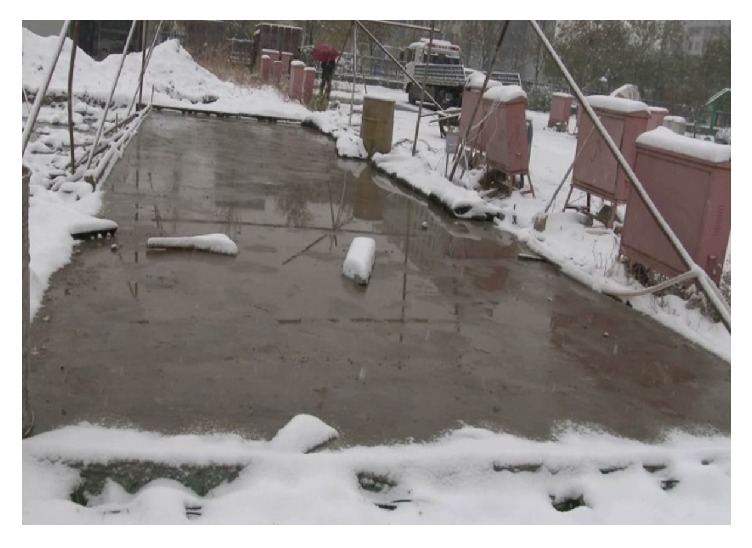
Snow melting performance of heat pipe pavement on 22 November 2015.

It was heavily snowing suddenly in Beijing on 22 November 2015. The snow started at 9:00 a.m. and stopped at 17:00, and the total thickness of snowfall on normal pavement was more than 10 cm. Hundreds of flights were canceled at Beijing Capital Airport on this day. In the snowfall time the air temperature is about −4°C, and the surface temperature of heat pipe pavement is about 2°C. As shown in Figure 10, the heat pipe snow melting pavement melted the snow completely at any time, which is secure enough for planes take-off and landing [24].

**Figure 11:**
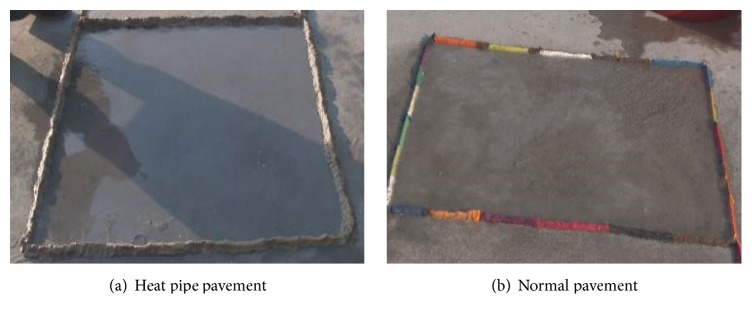
Antifreezing performance of heat pipe pavement on 10 February 2016.

The antifreezing performance between heat pipe pavement and normal pavement is compared Figure 11. The ice was made by the mixture of ice and water [25], and the thickness is 6 mm. As the result shows in Figure 11(a), the mixture of ice and water on heat pipe pavement surface changed to flowing water. However, the mixture of ice and water changed to hard ice after 3 minutes on the normal pavement surface shown in Figure 11(b), which is pretty dangerous for fight. The surface temperatures of heat pipe pavement and normal pavement were monitored as about 2°C and −5°C separately, and the environment temperature was −6°C. The snow melting and antifreeze performance of heat pipe pavement can strongly prove that utilizing heat pipe technology to melt snow and ice on the pavement is entirely feasible.

### 3.4. Energy Source of Heat Pipe Pavement Snow Melting

**Figure 12:**
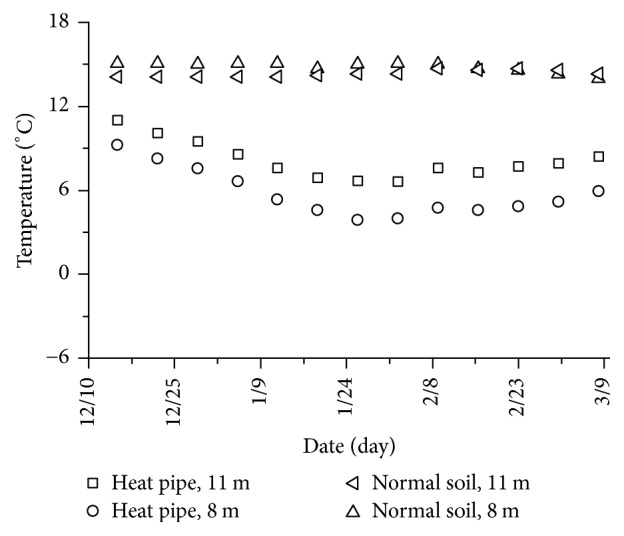
Ground soil temperature of heat pipe and normal soil.

The temperatures of the heat pipe and normal ground soil at 8 m and 11 m are shown in Figure 12. Due to the heat absorbed by the heat pipe evaporator, the temperature of heat pipe was lower than the normal soil at both 8 m and 11 m. The temperature of the heat pipe was decreased in winter, and the heat was recovered after 25 January, because the environment temperature increased gradually and the heat pipe system stopped working. It should be noted that the ranges of the temperature change at 8 m and 11 m of the heat pipe were nearly same in winter because of the quite high heat transfer of heat pipe system. Therefore, the ground soil can heat the pavement with the heat pipe system in winter and recover in summer by itself; this snow melting system can run for a long time.

## 4. 4. Conclusions

As a new snow melting and deicing technology on runway, heat pipe snow melting system was researched in this paper. The energy used in the system is the ground thermal energy which is the renewable and environmental friendly energy. In the present work, an actual full scale snow melting system with heat pipe technology is built, and experiments of the melting process are analyzed on the concrete pavement. From the snow melting system experimental results, the following conclusions can be obtained:

1. As the heat transfer effect of heat pipe system, the pavement temperature increases about 8°C, and the daily range of the pavement temperature decreases to 40%, which gives a more steady state to concrete.
2. The surface temperature decreases about 2°C as the pipe embedded depth increases from 6 cm to 15 cm; therefore, the pavement temperature field can be improved by a proper burial depth of heat pipe.
3. Environment temperature is a decision variable that has a dominant role in the pavement temperature field. It is probably freezing at heat pipe pavement when the air temperature is below −8°C.
4. Snow melting and antifreezing test result proves the well working performance of heat pipe snow melting system. This system is useful for melting sudden snowfall, which can ensure flight safety.
5. The ground soil heats the pavement with the heat pipe system in winter and recovers in summer by itself, and this snow melting system can run for a long time.
